# Supplementary figures and images for: TMC7 deficiency causes acrosome biogenesis defects and male infertility in mice
Source: eLife. 2024 Sep 13;13:RP95888. doi: 10.7554/eLife.95888 (PMC11398861; doi:10.7554/eLife.95888)

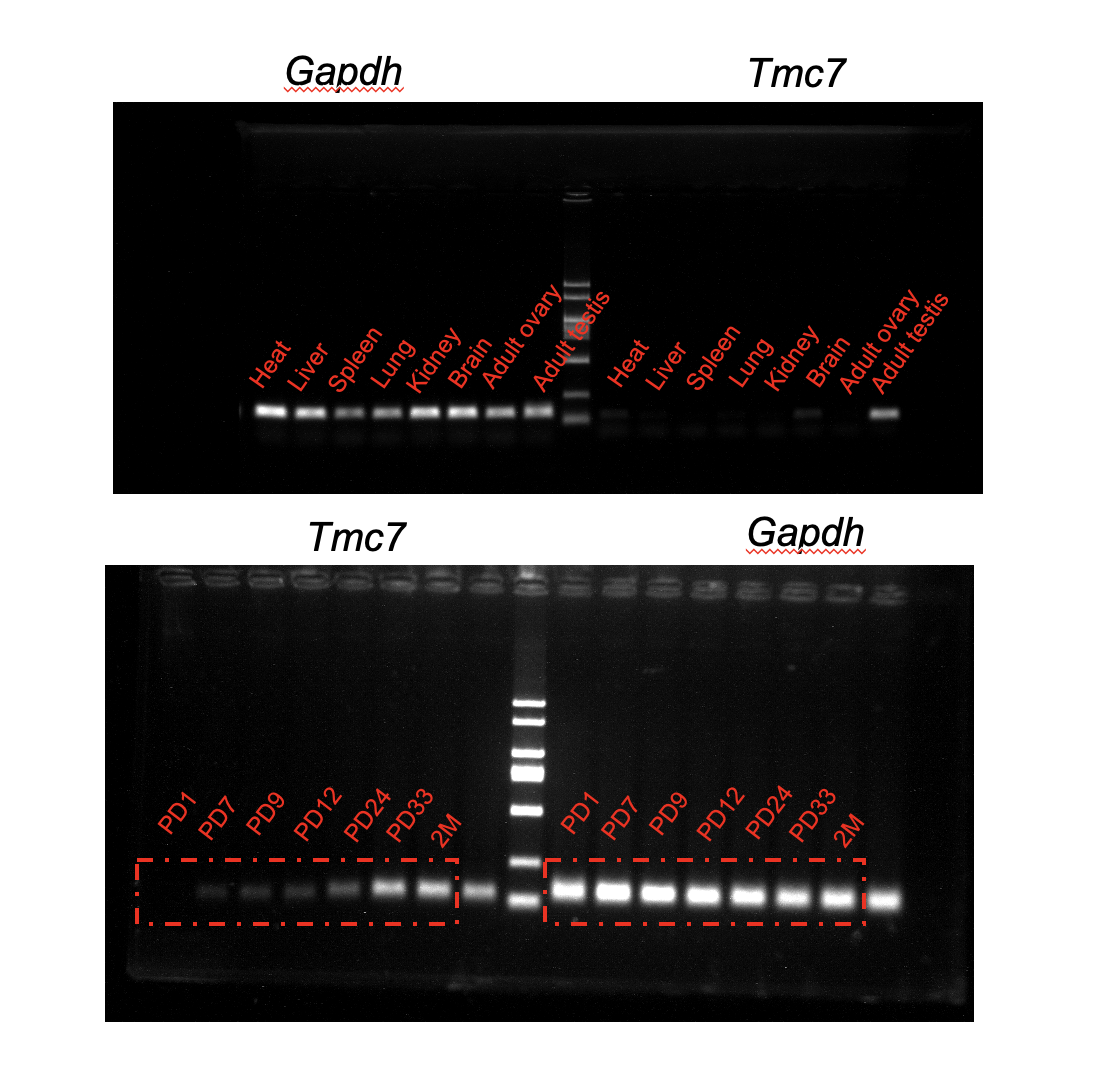

Supplement: Figure 1—source data 1. [file elife-95888-fig1-data1.zip › Uncropped and labeled gels for Figure 1.png]

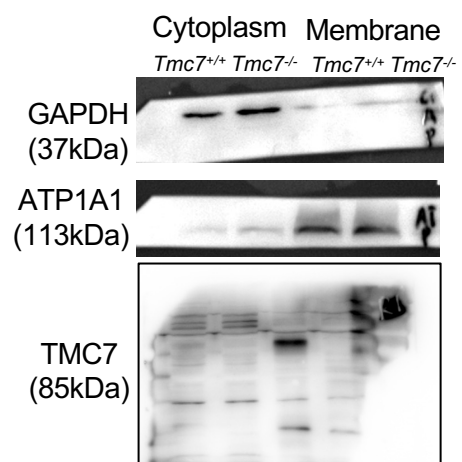

Supplement: Figure 1—figure supplement 1—source data 1. [file elife-95888-fig1-figsupp1-data1.zip › Figure 1-figure supplement 1-source data 1/uncropped and labelled gels for Figure1-figure supplement1 .pdf]

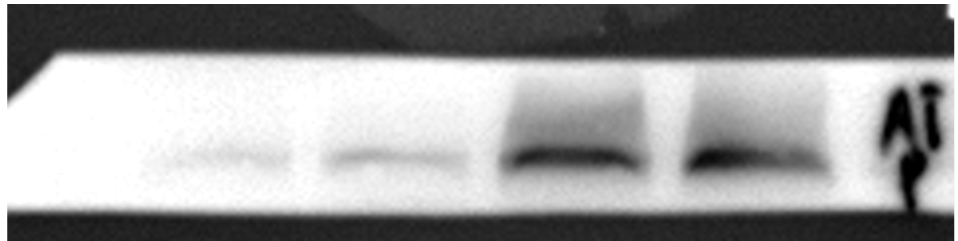

Supplement: Figure 1—figure supplement 1—source data 2. [file elife-95888-fig1-figsupp1-data2.zip › Figure 1-figure supplement 1-source data 2/Raw unedited gels ATP1A1 for Figure 1 -figure supplement 1 .png]

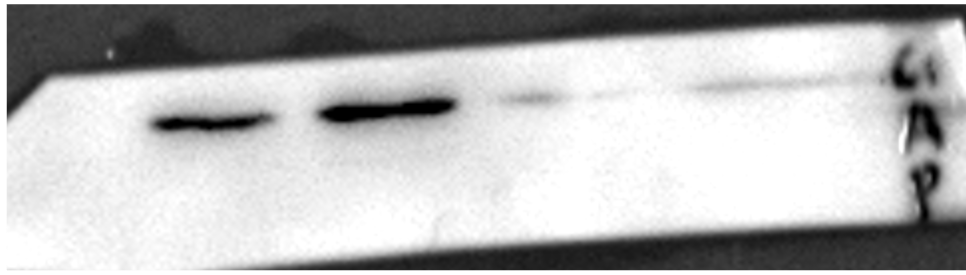

Supplement: Figure 1—figure supplement 1—source data 2. [file elife-95888-fig1-figsupp1-data2.zip › Figure 1-figure supplement 1-source data 2/Raw unedited gels GAPDH for Figure 1 -figure supplement 1.png]

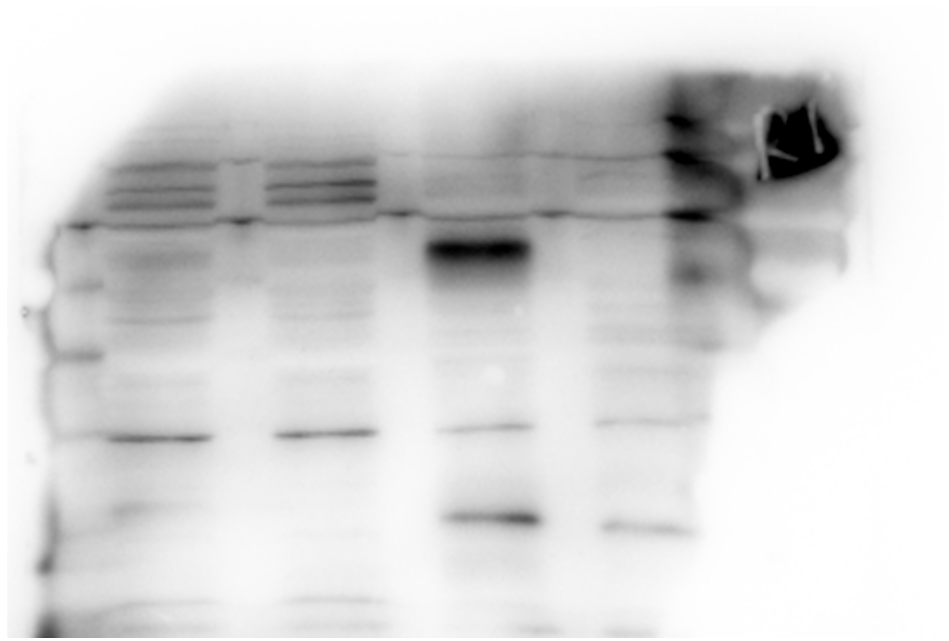

Supplement: Figure 1—figure supplement 1—source data 2. [file elife-95888-fig1-figsupp1-data2.zip › Figure 1-figure supplement 1-source data 2/Raw unedited gels TMC7 for Figure 1 -figure supplement 1 .png]

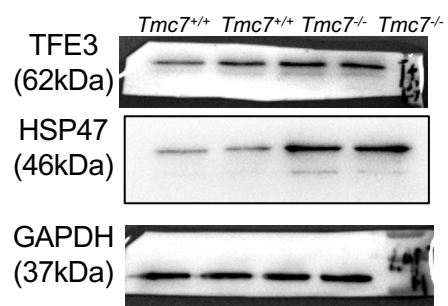

Supplement: Figure 3—source data 1. [file elife-95888-fig3-data1.zip › Figure 3-source data 1/Uncropped and labelled gels for Figure 3.pdf]

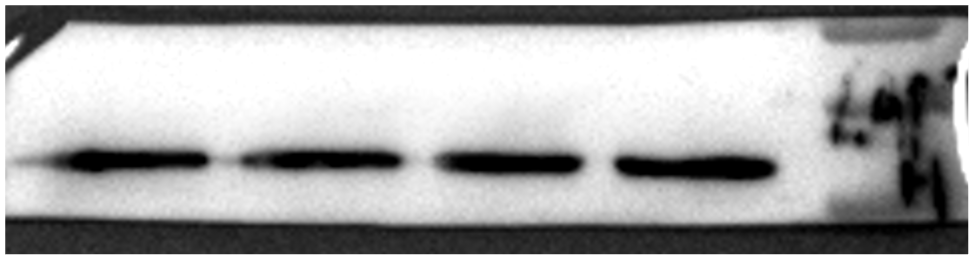

Supplement: Figure 3—source data 2. [file elife-95888-fig3-data2.zip › Figure 3-source data 2/Raw unedited gels GAPDH for Figure 3.png]

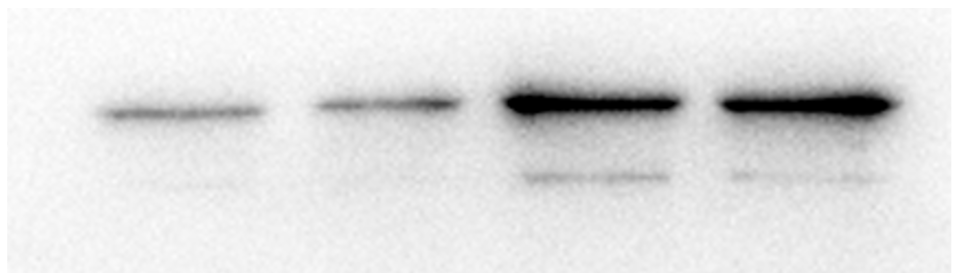

Supplement: Figure 3—source data 2. [file elife-95888-fig3-data2.zip › Figure 3-source data 2/Raw unedited gels HSP47 for Figure 3.png]

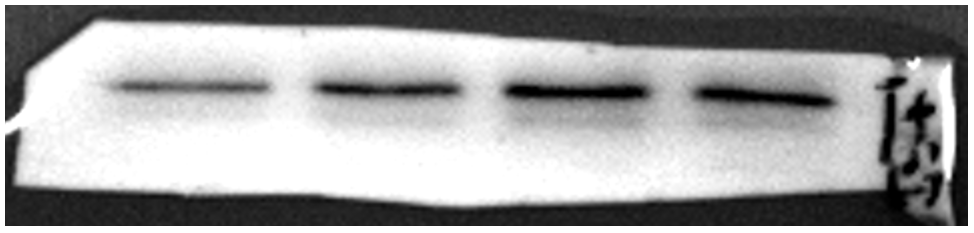

Supplement: Figure 3—source data 2. [file elife-95888-fig3-data2.zip › Figure 3-source data 2/Raw unedited gels TFE3 for Figure 3.png]

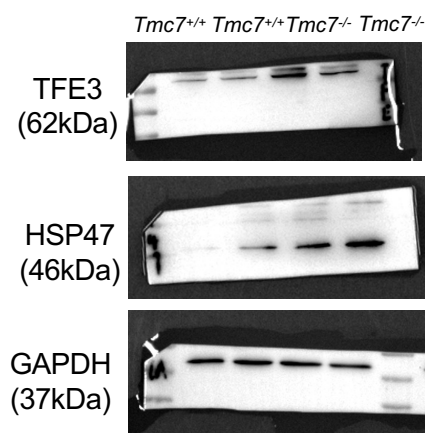

Supplement: Figure 3—figure supplement 1—source data 1. [file elife-95888-fig3-figsupp1-data1.zip › Figure 3-figure supplement 1-source data 1/Uncropped and labelled gels for Figure 3-figure supplement 1.pdf]

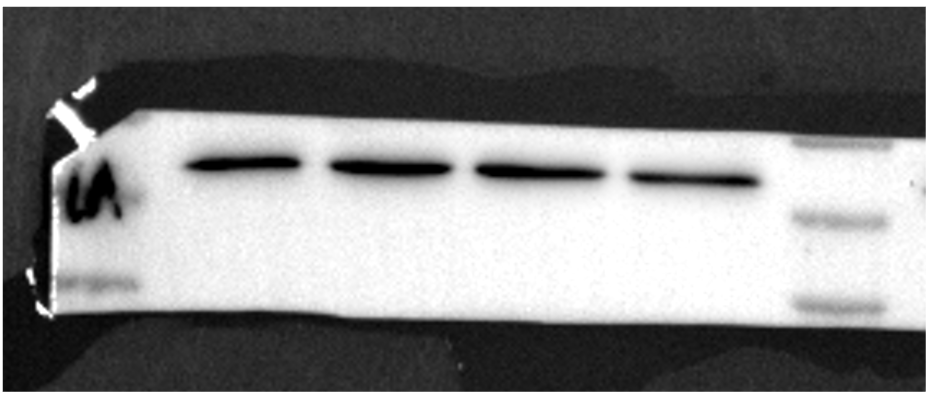

Supplement: Figure 3—figure supplement 1—source data 2. [file elife-95888-fig3-figsupp1-data2.zip › Figure 3-figure supplement 1-source data 2/Raw unedited gels GAPDH for figure 3-figure supplement 1.png]

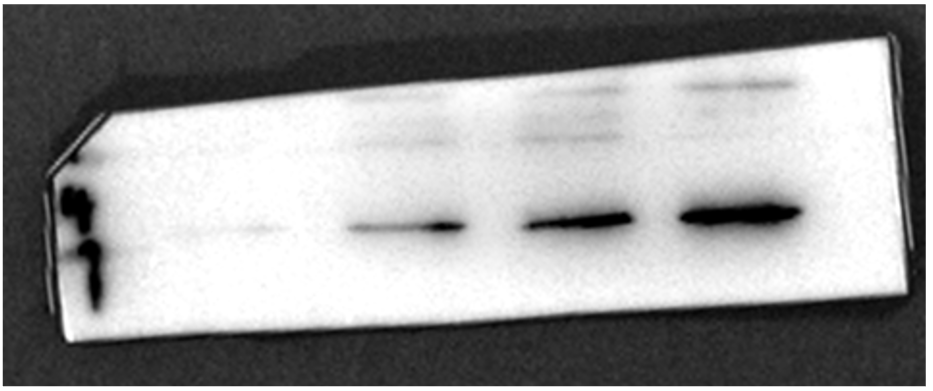

Supplement: Figure 3—figure supplement 1—source data 2. [file elife-95888-fig3-figsupp1-data2.zip › Figure 3-figure supplement 1-source data 2/Raw unedited gels HSP47 for Figure 3-figure supplement 1.png]

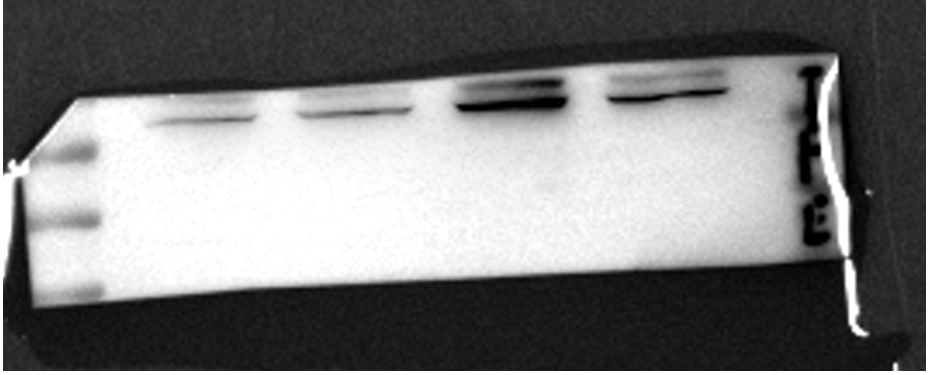

Supplement: Figure 3—figure supplement 1—source data 2. [file elife-95888-fig3-figsupp1-data2.zip › Figure 3-figure supplement 1-source data 2/Raw unedited gels TFE3 for Figure 3-figure supplement 1.png]

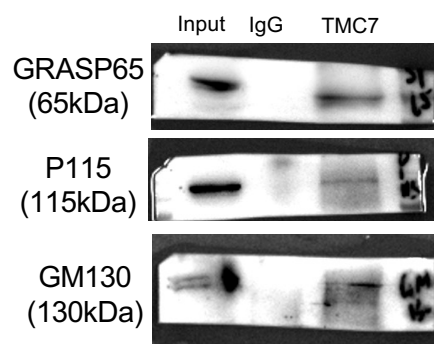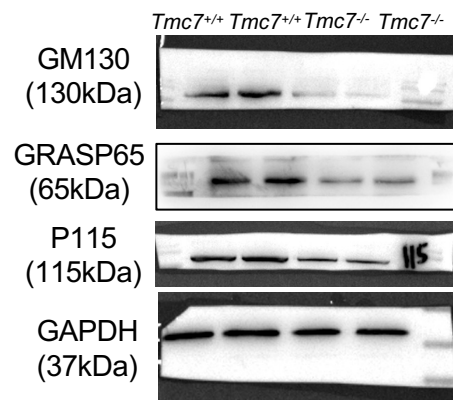

Supplement: Figure 4—source data 1. [file elife-95888-fig4-data1.zip › Figure 4-source data 1/uncropped and labelled gels for figure 4.pdf]

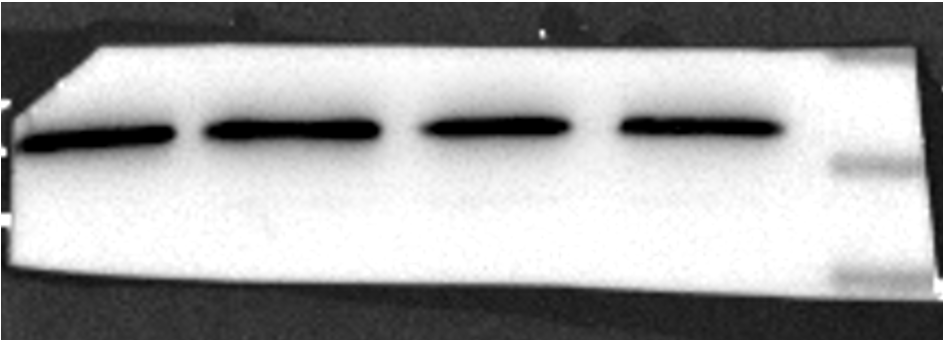

Supplement: Figure 4—source data 2. [file elife-95888-fig4-data2.zip › Figure 4-source data 2/Raw unedited gels GAPDH for Figure 4.png]

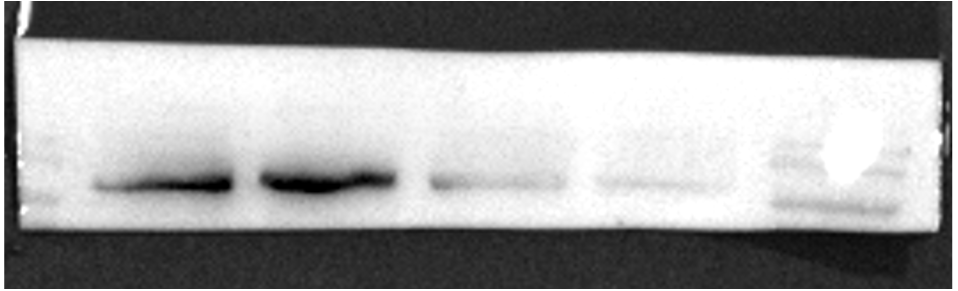

Supplement: Figure 4—source data 2. [file elife-95888-fig4-data2.zip › Figure 4-source data 2/Raw unedited gels GM130 for Figure 4.png]

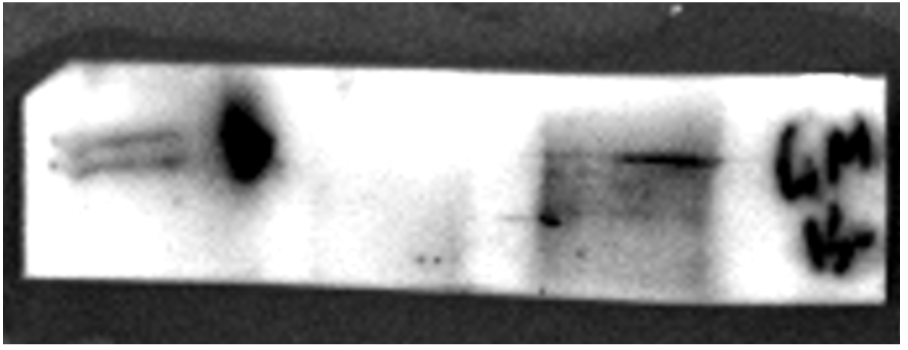

Supplement: Figure 4—source data 2. [file elife-95888-fig4-data2.zip › Figure 4-source data 2/Raw unedited gels GM130(co-IP) for Figure 4.png]

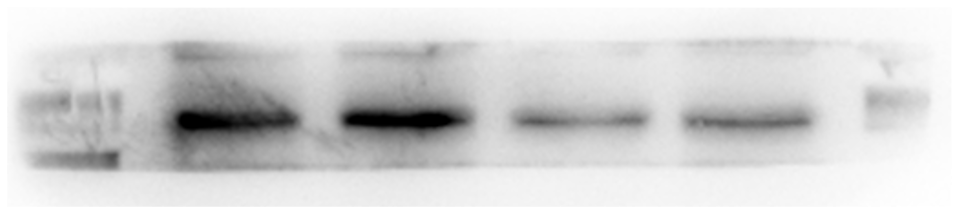

Supplement: Figure 4—source data 2. [file elife-95888-fig4-data2.zip › Figure 4-source data 2/Raw unedited gels GRASP65 for Figure 4.png]

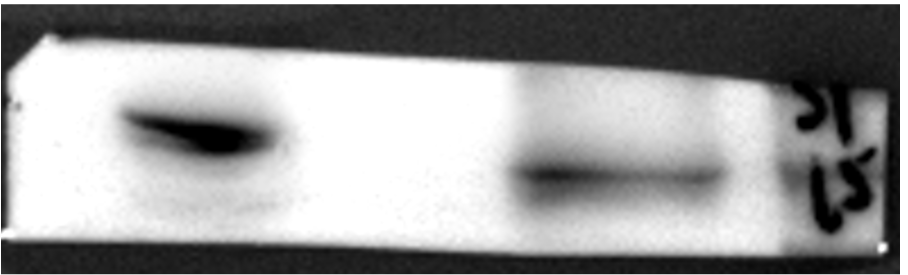

Supplement: Figure 4—source data 2. [file elife-95888-fig4-data2.zip › Figure 4-source data 2/Raw unedited gels GRASP65(co-IP) for Figure 4.png]

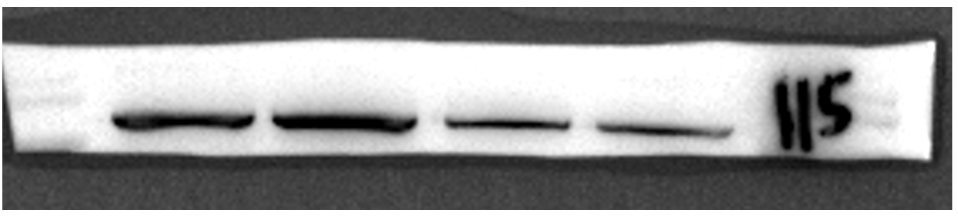

Supplement: Figure 4—source data 2. [file elife-95888-fig4-data2.zip › Figure 4-source data 2/Raw unedited gels P115 for Figure 4.png]

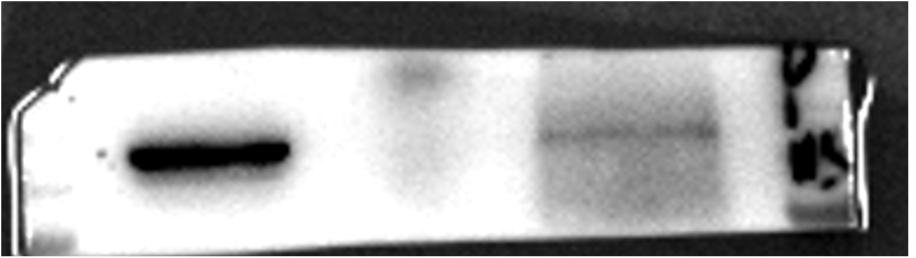

Supplement: Figure 4—source data 2. [file elife-95888-fig4-data2.zip › Figure 4-source data 2/Raw unedited gels P115(co-IP) for Figure 4.png]

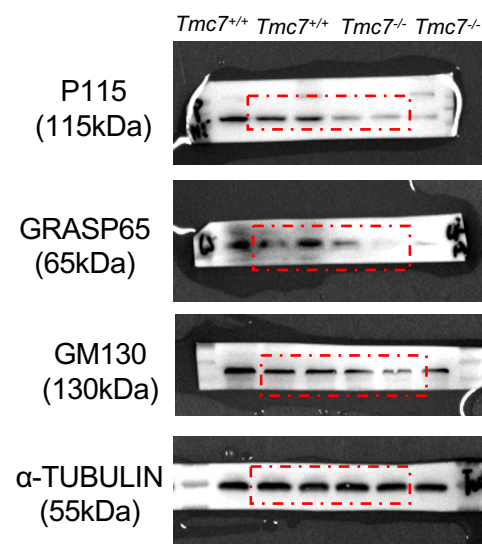

Supplement: Figure 4—figure supplement 1—source data 1. [file elife-95888-fig4-figsupp1-data1.zip › Figure 4-figure supplement 1-source data 1/Uncropped and labelled gels for Figure 4-figure supplement 1.pdf]

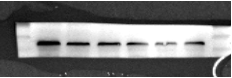

Supplement: Figure 4—figure supplement 1—source data 2. [file elife-95888-fig4-figsupp1-data2.zip › Figure 4-figure supplement 1-source data 2/Raw unedited gels GM130 for Figure 4-figure supplement 1.png]

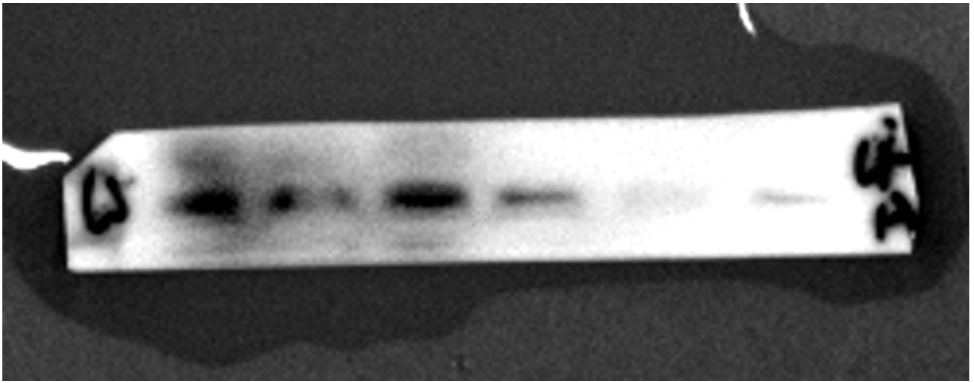

Supplement: Figure 4—figure supplement 1—source data 2. [file elife-95888-fig4-figsupp1-data2.zip › Figure 4-figure supplement 1-source data 2/Raw unedited gels GRASP65 for Figure 4-figure supplement 1.png]

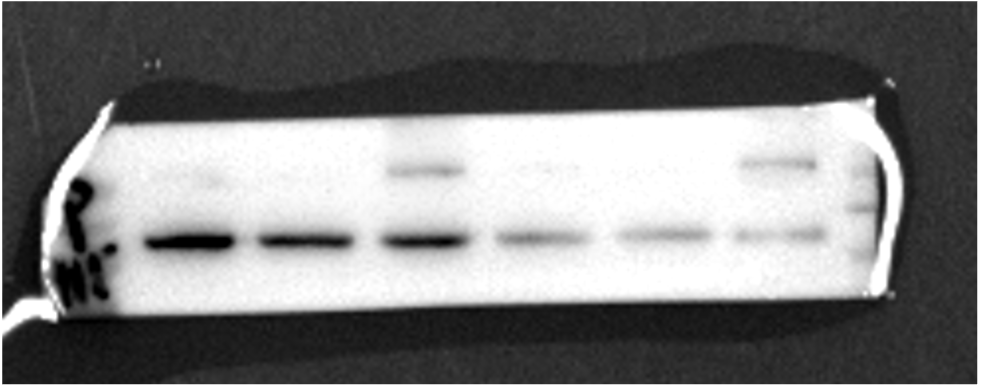

Supplement: Figure 4—figure supplement 1—source data 2. [file elife-95888-fig4-figsupp1-data2.zip › Figure 4-figure supplement 1-source data 2/Raw unedited gels P115 for Figure 4-figure supplement 1.png]

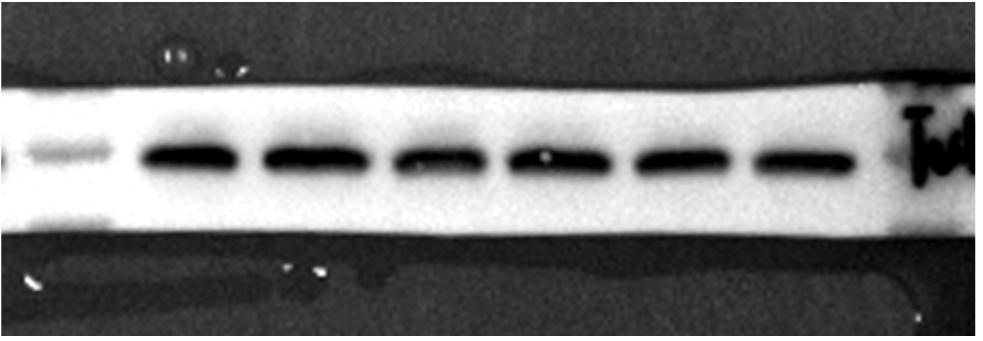

Supplement: Figure 4—figure supplement 1—source data 2. [file elife-95888-fig4-figsupp1-data2.zip › Figure 4-figure supplement 1-source data 2/Raw unedited gels tubulin for Figure 4-figure supplement 1.png]

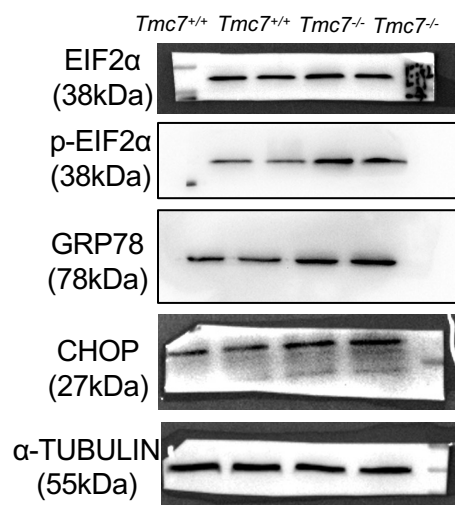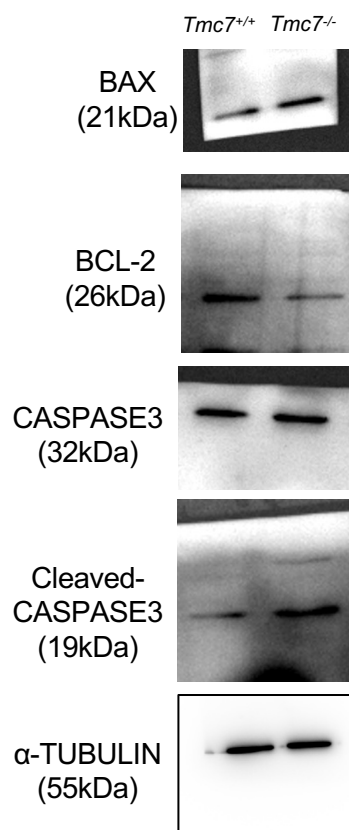

Supplement: Figure 5—source data 1. [file elife-95888-fig5-data1.zip › Figure 5-source data 1/uncropped and labelled gels for Figure 5.pdf]

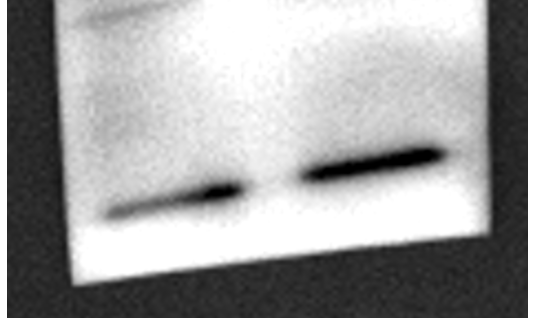

Supplement: Figure 5—source data 2. [file elife-95888-fig5-data2.zip › Figure 5-source data 2/Raw unedited gels BAX for Figure 5.png]

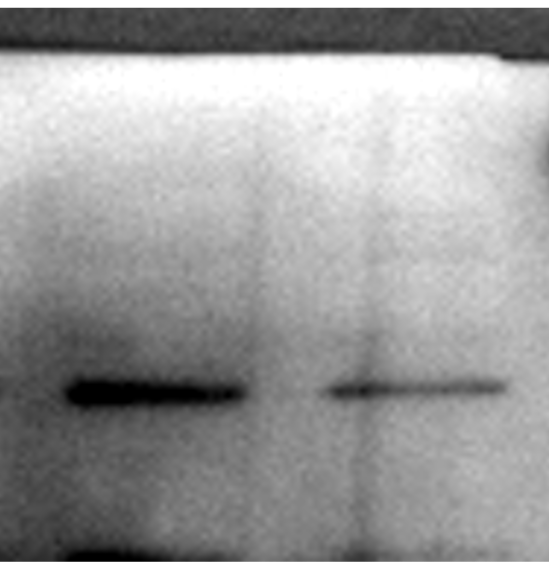

Supplement: Figure 5—source data 2. [file elife-95888-fig5-data2.zip › Figure 5-source data 2/Raw unedited gels BCL2 for Figure 5.png]

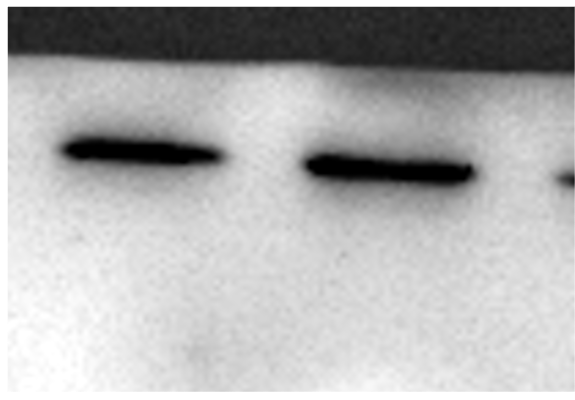

Supplement: Figure 5—source data 2. [file elife-95888-fig5-data2.zip › Figure 5-source data 2/Raw unedited gels CASPASE3 for Figure 5.png]

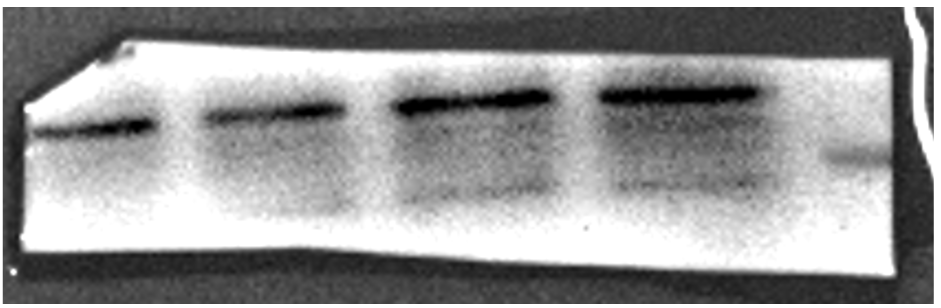

Supplement: Figure 5—source data 2. [file elife-95888-fig5-data2.zip › Figure 5-source data 2/Raw unedited gels CHOP for Figure 5.png]

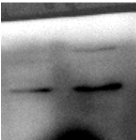

Supplement: Figure 5—source data 2. [file elife-95888-fig5-data2.zip › Figure 5-source data 2/Raw unedited gels CLEAVED-CASPASE3 for Figure 5.png]

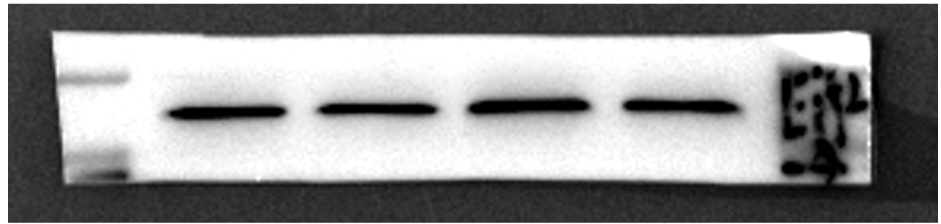

Supplement: Figure 5—source data 2. [file elife-95888-fig5-data2.zip › Figure 5-source data 2/Raw unedited gels EIF2a for Figure 5.png]

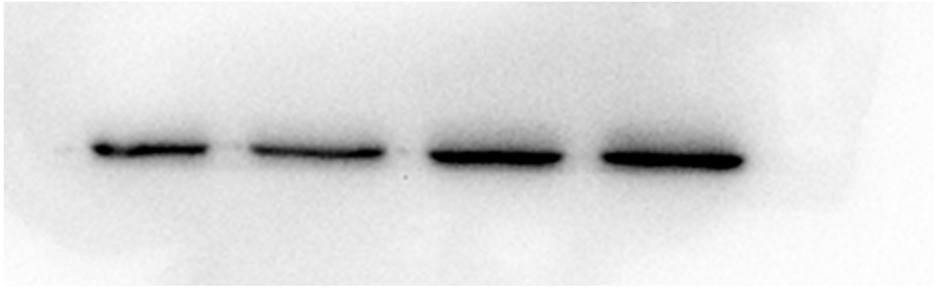

Supplement: Figure 5—source data 2. [file elife-95888-fig5-data2.zip › Figure 5-source data 2/Raw unedited gels GRP78 for Figure 5.png]

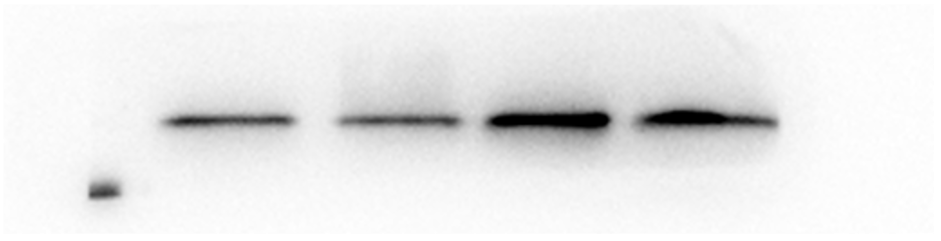

Supplement: Figure 5—source data 2. [file elife-95888-fig5-data2.zip › Figure 5-source data 2/Raw unedited gels p-EIF2a for Figure 5.png]

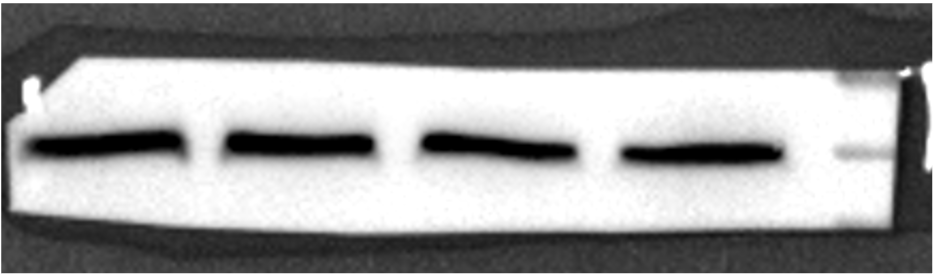

Supplement: Figure 5—source data 2. [file elife-95888-fig5-data2.zip › Figure 5-source data 2/Raw unedited gels TUBULIN for Figure 5.png]

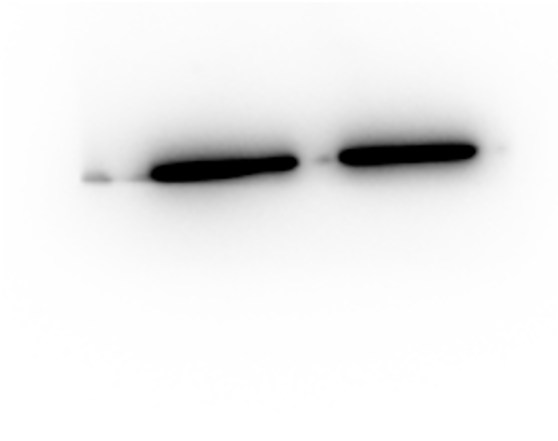

Supplement: Figure 5—source data 2. [file elife-95888-fig5-data2.zip › Figure 5-source data 2/Raw unedited gels TUBULIN-2 for Figure 5-source data 15 .png]

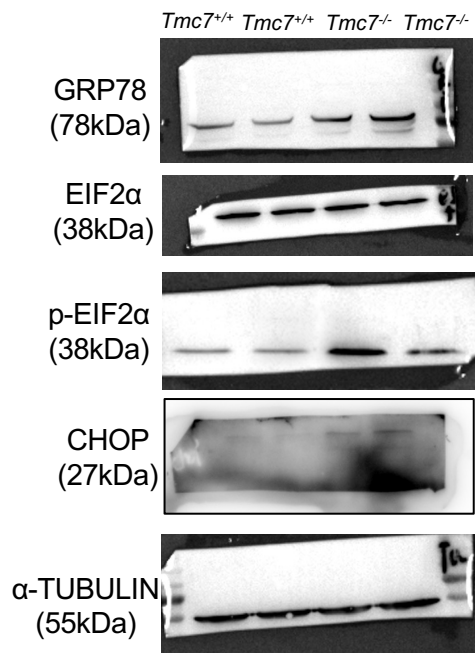

Supplement: Figure 5—figure supplement 1—source data 1. [file elife-95888-fig5-figsupp1-data1.zip › Figure 5-supplement figue1 source data 1/uncropped and labelled gels for figure5-supplemenr figure1.pdf]

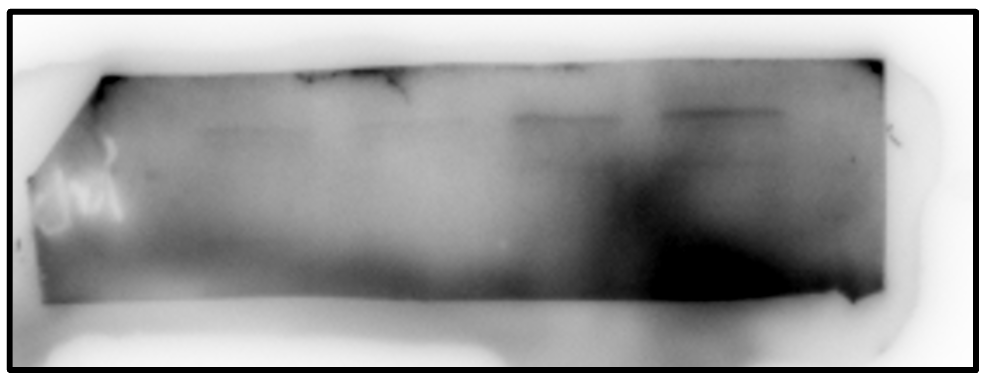

Supplement: Figure 5—figure supplement 1—source data 2. [file elife-95888-fig5-figsupp1-data2.zip › Figure 5-supplement figue1 source data 2/Raw unedited gels CHOP for Figure 5-figure supplement 1.png]

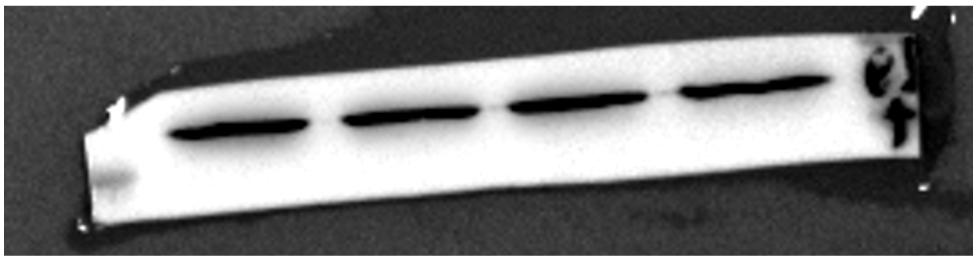

Supplement: Figure 5—figure supplement 1—source data 2. [file elife-95888-fig5-figsupp1-data2.zip › Figure 5-supplement figue1 source data 2/Raw unedited gels EIF2a for Figure 5-figure supplement 1.png]

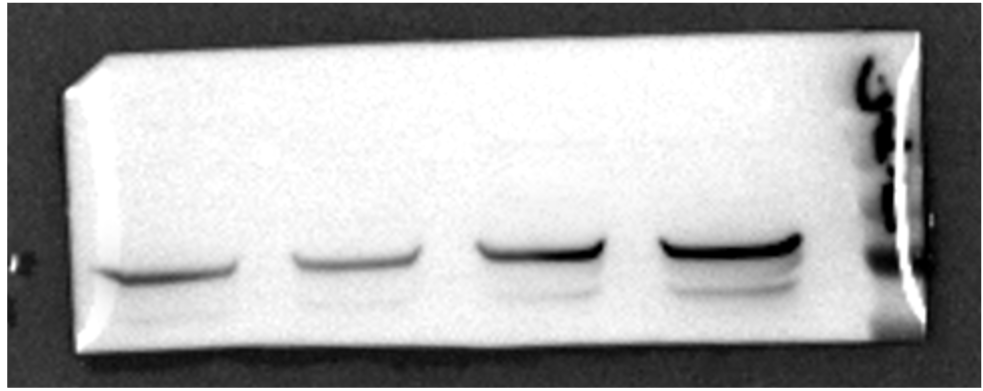

Supplement: Figure 5—figure supplement 1—source data 2. [file elife-95888-fig5-figsupp1-data2.zip › Figure 5-supplement figue1 source data 2/Raw unedited gels GRASP65 for Figure 5-figure supplement 1.png]

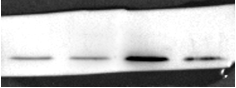

Supplement: Figure 5—figure supplement 1—source data 2. [file elife-95888-fig5-figsupp1-data2.zip › Figure 5-supplement figue1 source data 2/Raw unedited gels p-EIF2afor Figure 5-figure supplement 1.png]

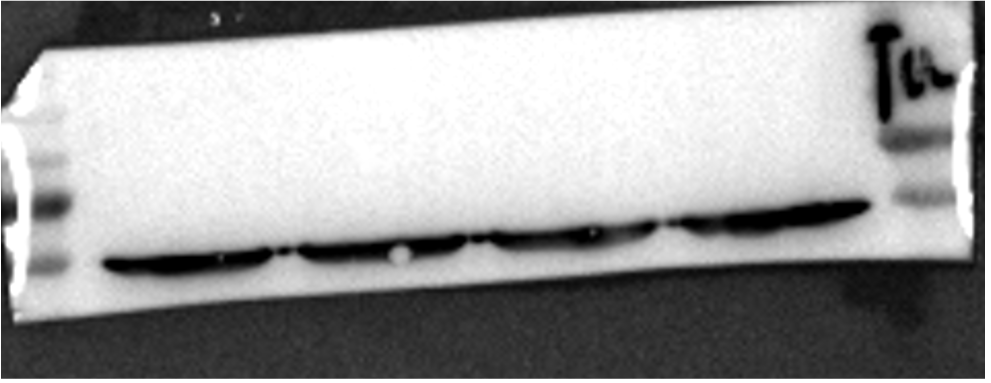

Supplement: Figure 5—figure supplement 1—source data 2. [file elife-95888-fig5-figsupp1-data2.zip › Figure 5-supplement figue1 source data 2/Raw unedited gels tubulin for Figure 5-figure supplement 1.png]
