# Supplementary material for: TMC7 deficiency causes acrosome biogenesis defects and male infertility in mice
Source: eLife. 2024 Sep 13;13:RP95888. doi: 10.7554/eLife.95888 (PMC11398861; doi:10.7554/eLife.95888)
Supplement: Supplementary file 1. [file elife-95888-supp1.docx]

Table1. Fertility testing of *Tmc7*^+/-^, *Tmc7*^-/-^ male mice and *Tmc7*^-/-^ female mice.

| **Male** | **Female** | **Num of pups/litter** |
| --- | --- | --- |
| *Tmc7^-/-^* | WT * 2 | None |
| *Tmc7^-/-^* | WT * 2 | None |
| *Tmc7^-/-^* | WT * 2 | None |
| *Tmc7^+/-^* | *Tmc7^+/-^* * 2 | 8,5,6,8 |
| *Tmc7^+/-^* | *Tmc7^-/-^* * 2 | 9,10,11,8 |
| *Tmc7^+/-^* | *Tmc7^-/-^* * 2 | 8,6,5,8 |
